# Influence of mental health and physical activity on memory complaints in older adults: results from the HUNT study

**DOI:** 10.1186/s12877-026-07015-7

**Published:** 2026-02-24

**Authors:** Skender Elez Redzovic, Mehdi Moloudi, Tore Bonsaksen

**Affiliations:** 1 Department of Neuromedicine and Movement Science, Faculty of Medicine and Health Sciences, NTNU Norwegian University of Science and Technology, S.P. Andersens veg 11, Trondheim, Norway; 2 Department of Health and Nursing Science, Faculty of Social and Health Science, University of Inland Norway, Elverum, Norway; 3 Department of Health, Faculty of Health Science, VID Specialized University, Stavanger, Norway

**Keywords:** Physical activity, Mental health, Subjective memory impairment, Short-term memory complaints, Long-term memory complaints, Gender, Older adults, Longitudinal study, Trøndelag health study (HUNT), Norway

## Abstract

**Background:**

Subjective memory impairment, including short-term (STM) and long-term (LTM) memory complaints, is common in older adults and may signal underlying cognitive decline. Good mental health (MH) and physical activity (PA) have been associated with better cognitive functioning and reduced risk of memory problems, yet their relationships with self-reported memory complaints remain ambiguous. Additionally, the impact of mid-adulthood MH and PA on later-life memory complaints is not well understood. Few large-scale population-based studies have examined these associations longitudinally.

**Aim:**

This population-based study has two primary aims: (a) to examine the concurrent associations between self-reported MH, PA, and subjective memory complaints in older adults, and (b) to investigate the longitudinal associations between mid-adulthood MH and PA and memory complaints 11 years later, with both analyses controlling for demographic factors.

**Methods:**

This mixed cross-sectional and longitudinal study used data from the third (2006–2008) and the fourth (2017–2019) waves of the HUNT Study in Norway. Subjective self-reported STM and LTM complaints were measured by the nine-item Meta-Memory Questionnaire. The sample included 9,226 and 6,986 healthy participants from the HUNT3 and HUNT4 waves, respectively, who completed the questionnaire and were aged 60 or above by HUNT4. Independent t-tests, ANOVAs and linear regression analysis were used to examine group differences and associations between PA, MH, and memory complaints.

**Results:**

While individuals with lower levels of PA had more STM complaints in both HUNT3 and HUNT4, these associations did not hold after adjustment for sociodemographic and mental health-related variables. However, male gender and MH problems – in particular depressive symptoms – were found to be related to LTM and STM complaints, concurrently and in part longitudinally.

**Conclusion:**

Male gender and MH - particularly depressive symptoms—emerged as consistently associated with both STM and LTM complaints in older adults, both concurrently and, to an extent, over time. Although PA initially appeared related to memory complaints, this association weakened after accounting for MH and sociodemographic factors. These findings highlight MH as a key modifiable factor in addressing memory complaints in aging. Future research should clarify the causal mechanisms linking depressive symptoms and subjective memory complaints.

## Introduction

The remarkable reduction in mortality over the past century has led to a rapid increase in the global population, with the 21st century anticipated to be the “century of aging” [1]. In Norway, this demographic shift is particularly notable, with a growing older adult population. By 2060, the proportion of people aged 70 and older is expected to rise from the current 11% to 19% [2, 3].

Aging brings physiological changes that can affect health and daily functioning in activities of daily living [4], including a reduced capacity to fight illness, increased physical limitations, and cognitive decline. In an increasingly complex world, the demands on cognitive functions are growing, placing significant pressure on mental processes that originally evolved to handle a much simpler environment [5]. Memory is one of the most significantly impacted cognitive functions during the aging process [6], leading to challenges in carrying out daily activities, planning and completing tasks, and maintaining effective communication skills [7]. Although memory impairment is common among older adults, only a small number of individuals tend to openly express concerns about their memory, unless prompted by their general physician [8]. This non-reporting may cause further difficulties in daily functioning [9].

Memory impairment can be measured using both objective and subjective methods. Objective memory impairment refers to measurable deficits in memory performance that can be identified through formal testing. Subjective memory impairment (SMI) refers to individuals’ self-assessment and recognition of memory decline [10]. However, definitions vary widely in terms of the types of questions used to determine SMI and additional features pertaining to memory complaints included in the definition [11]. Subjective memory is multidimensional [12–14]. It is composed of multiple functional systems, including short-term memory complaints (STM, also referred to as working memory) and long-term memory complaints (LTM). LTM is responsible for retaining information over extended periods, whereas STM holds information temporarily for immediate use [7, 15]. This distinction is essential for understanding how individuals assess and experience memory challenges. LTM is further divided into declarative memory, which encompasses episodic and semantic memory, and non-declarative memory, which includes procedural and perceptual memory. Additionally, three key processes—encoding, maintenance/consolidation, and retrieval—operate on these memory systems [15]. Episodic and working memory typically show the strongest age-related declines, whereas STM shows relatively minimal change. In contrast, semantic memory often remains stable or even improves with age [6].

Higher levels of SMI are associated with poorer ability to plan and perform tasks and communication skills, difficulties in daily activities, working life, overall well-being, and mortality risk [7, 9]. However, it is important to distinguish SMI from clinically diagnosed memory disorders, which are not normative and represent more severe cognitive impairments [6]. SMI is only weakly related to objective memory impairment [16]. In a recent study, subjective memory complaints were reported by 42% of the sample, whereas objective memory impairment was present in only one-quarter of these individuals [17]. This indicates that most people with memory complaints do not exhibit objective memory deficits. However, memory-related disorders contribute to the economic burden. For instance, in Norway caring for the older adults needs more than 25% of the municipal budget which is almost 3% of the GDP [18]. Studying SMI is crucial because it can serve as an early indicator of cognitive decline, enabling timely screening and intervention for conditions ranging from mild cognitive impairment to severe dementia. Understanding SMI helps identify individuals at risk before objective deficits appear and guides the development of strategies to improve cognitive function, daily functioning, quality of life, and mental well-being. It also informs clinical decision-making and the allocation of healthcare resources, even when memory performance remains within normal limits [19].

Physical activity (PA) is one of the key behavioral strategies known to enhance memory and cognitive function. Regular PA has been shown to benefit brain health by helping to maintain and improve memory performance, and it is widely recognized as a modifiable risk factor for dementia [5, 20, 21]. PA is defined as any bodily movement produced by skeletal muscles that requires energy (World Health Organization, 2022). However, existing research leaves some knowledge gap regarding the relationship between PA and SMI. First, while several studies have demonstrated a positive correlation between PA and memory performance [7, 22–25], others have not observed such associations [20, 26]. Most of these investigations have relied on objective methods to measure PA which have limitations such as small sample sizes and the alteration of participants’ natural environments or activities which may affect their levels of PA.

Second, the nature of the relationship between PA in early life with SMI at old age is not fully established. Youth who engage in regular PA tend to exhibit better cognitive and academic performance [27]. It is possible that, much like early-life education, early-life PA could contribute to building better “cognitive reserve” with long-lasting benefits. Studies suggest that being active over the life course lowers likelihood of cognitive impairment in late life [28–31]. However, the effect of PA on LTM and STM complaints are not explored. A recent study [32] demonstrated that PA during early and mid-adulthood independently predicted both higher episodic memory levels and a slower rate of memory decline in later life, through improvements in the cardiovascular system, particularly blood pressure control. To our knowledge there are no epidemiological studies that address the relationship between PA in mid-adulthood and STM and LTM complaints in later life.

Furthermore, MH symptoms, including depression, anxiety, and psychological distress, are strong correlates of memory complaints and can also influence PA patterns [5, 33–37]. Some researchers suggest that subjective memory complaints may serve as an early indicator of dementia [33, 34, 36, 38, 39]. Subjective memory complaints are linked to depression and anxiety in cross-sectional studies, and longitudinal research shows they can precede dementia [40]. SMI represents a preclinical indicator of neurocognitive decline, supporting early diagnostic stratification and intervention in mild cognitive impairment, a transitional condition bridging healthy aging and dementia. Notably, recent evidence highlights PA and depression as modifiable risk factors for dementia [21, 41]. However, longitudinal population-based studies on the association between SMI, PA and MH symptoms remain scarce.

Several demographic and health-related factors are independently associated with both PA, MH, and SMI, potentially biasing observed associations if not properly accounted for. Age is one of the strongest predictors of SMI, with older adults reporting more frequent memory complaints [42, 43]. Gender differences have also been observed, with some studies suggesting that women have higher rates of SMI despite performing similarly or better than men on objective memory tests [42, 44, 45]. Moreover, good quality education for all, as well as cognitively stimulating activities in midlife also protect cognition [21]. BMI and **s**moking behavior are other important factors; smoking is known to negatively affect both cardiovascular and cognitive health and may confound the relationship between PA and memory outcomes [21, 46–48]. Additionally, living situation—such as living alone versus with others—can influence both MH and opportunities for engaging in PA [42]. Sleep quality, particularly insomnia, is closely tied to memory function and cognitive performance, and poor sleep is often associated with both lower PA and higher SMI [49]. Neglecting to control for these factors can result in misleading conclusions about the role of PA and MH in memory performance.

Given the identified gaps in the existing literature, the aim of this study is to utilize data from the Trøndelag Health Study (HUNT) to explore (a) concurrent associations between PA, MH and both subjective STM and LTM complaints at two different time points; and (b) longitudinal associations between mid-adulthood PA and MH and subjective memory complaints 11 years later. We hypothesize positive associations in both cases.

## Methods

The data for this mixed cross-sectional and longitudinal study was collected from older, community-dwelling adults in Trøndelag county, Norway, as part of the third and fourth wave of the Trøndelag Health Study (HUNT). The population in Trøndelag county is considered representative of the Norwegian population [50]. The HUNT3 (2006–2008) and HUNT4 (2017–2019) study had a high participation rate, with 56,042 participants (54.0%) from North Trøndelag and 107,711 participants (42.6%) from South Trøndelag, minimizing the risk of selection bias. However, participation rates were slightly higher among individuals aged 40–79 years and lower among those who were unmarried or living in urban areas. The sample was also ethnically homogeneous, which limits the generalizability of the findings to individuals from non-European backgrounds [50]. Despite these limitations, the HUNT3 and HUNT4 cohorts provide a valuable resource for studying health and cognitive outcomes in a large, representative population of older adults in Norway.

### Participants

In the HUNT3 study, out of 93,860 eligible individuals aged 20 or older who were invited to participate, 50,807 (54.1%) completed the questionnaire. Among these respondents, 27,758 were female and 23,049 were male [51]. In HUNT4, of 103,800 invitees, 56,042 took part by returning the questionnaire. This included 30,574 women and 25,468 men. During the transition from HUNT3 to HUNT4, 7,763 individuals died or moved and 9,218 did not participate in HUNT4. Finally, 33,818 individuals were participants in both HUNT3 and HUNT4.

Our study focused exclusively on those aged 60 years or older by the time of HUNT4 (born in 1958 or before) and who also participated in HUNT3. Although age-related declines in cognitive functioning, including memory, begin in early adulthood, memory decline tends to accelerate around the age of 60, making this age group particularly relevant for the present analyses [6]. The exclusion of participants with medical conditions that restricted their PA was necessary to minimize confounding that could potentially affect the results. To identify these participants, we used the question, “Do you suffer from longstanding (at least 1 year) illness or injury of a physical or psychological nature that impairs your functioning in your daily life?” with two possible answers of “No” and “Yes”. Additionally, the presence of conditions such as angina (chest pain), asthma, arthritis and spondylarthritis were criteria for exclusion. After excluding participants based on these criteria, 9,226 individuals participated in the 2006–2008 wave (HUNT3), 6,986 participated in the 2017–2019 wave (HUNT4), and 5,100 individuals participated in both waves. Individuals with missing data on the variables employed in the analytic procedures were excluded casewise (analysis-by-analysis).

### Measures

#### Physical activity

We obtained data on PA through the self-reported HUNT study questionnaire. The questions used to assess PA has demonstrated strong reliability, with high correlations and good to very good kappa statistics observed between test and retest [52]. The first question assesses how frequently participants took part in PA during a typical week. They were given five response options: (1) never, (2) less than once a week, (3) once a week, (4) 2–3 times a week and (5) nearly every day. For our study, we recoded PA frequency to the values indicating the number of occurrences per week (0, 0.5, 1, 2.5, 5). The second question focuses on the average intensity of PA, with three response options: (1) I take it easy, I don’t get out of breath or break a sweat, (2) I push myself until I’m out of breath and break into a sweat (3) I practically exhaust myself. These responses remained without change as reported in the questionnaire (1, 2 and 3 indicating light, moderate and vigorous activity, respectively). Based on the study by [53], the frequency and intensity measurements were combined to create a single PA index. The PA index scores, theoretically ranging from 0 to 15, were divided into three categories using the 33rd and 67th percentiles as cutoff points: low (< 2.5), intermediate (2.5-5.0) and high (> 5.0) levels of PA.

#### Subjective memory

Subjective memory impairment, including LTM and STM complaints, was examined by using the Metamemory Questionnaire (MMQ). MMQ was originally designed for NORA-study of individuals aged 30–69 and + 70 [54]. The questionnaire consists of nine items related to memory challenges. The first two concern overall memory capacity: 1) Do you have problems with your memory? and 2) Has your memory changed since you were younger?“. Response options were “No”, “Yes, some” and “Yes, a lot”. The next seven items assess specific memory functions. Four of them focus on recalling recent events or activities: 3) Do you have trouble remembering things that happened a few minutes ago?, 6) Do you have trouble remembering to do something you have planned to do?, 7) Do you have trouble remembering things that happened a few days ago?, and 9) Do you have trouble keeping track of a conversation? Three items are about remembering previous information: 4) Do you have trouble remembering other people’s names?, 5) Do you have trouble remembering dates?, and 8) Do you have trouble remembering things that happened years ago? Response options were “never,” “sometimes,” or “often” for each of these questions. In line with earlier studies using the MMQ, such as the studies by Moradi et al. and Almkvist et al. [10, 15], it was determined that items 1, 2, 4, 5, and 8 are indicative of LTM complaints, while questions 3, 6, 7, and 9 are indicative of STM complaints. Scoring was based on the methodology of Moradi et al. [10]. For questions 1 and 2 we assigned a score of 0 for ‘No’, 1 for ‘Yes, sometimes’, and 2 for ‘Yes, a lot’. For questions 3–9, the assigned scores were 1 for “Never”, 2 for “Sometimes” and 3 for “Often”. After summing the scores, scores ranged 3–13 for LTM and 4–12 for STM complaints. The scores of 3 for LTM and 4 for STM complaints indicated no memory impairment, while scores of 13 for LTM and 12 for STM complaints indicated the highest level of memory problems.

#### Anxiety and depression symptoms

In the assessment of anxiety and depression, the Hospital Anxiety and Depression Scale (HADS) was used [55]. This scale has 14 items, with seven focusing on anxiety and seven on depression. Each item has four response options (0–3). Positively worded items are reverse coded before summing the scale scores. Scale scores ranging 0–7 on both the anxiety and depression scales were defined as indicating the absence of anxiety or depression, while scale scores of 8 or higher suggested the presence of anxiety or depression, respectively [56, 57].

#### Confounding variables

In this study, adjustment for a range of potentially confounding variables were made to ensure the integrity and validity of the findings. These variables included age, sex, education, living with a spouse or partner, body mass index (BMI), insomnia, anxiety, depression and smoking habits. For the initial presentation, age was divided into three categories: 60–69 years, 70–79 years, and 80 years or older, but the variable was kept as continuous in the multivariate analyses. Sex was recorded as male or female. The participants’ educational background was assessed with the question: “What is the highest level of education you have completed?” There were six options available: primary school, 1–2 years of upper secondary school, 3 years in upper secondary school, certificate of apprenticeship or journeyman’s certificate, college/university less than 4 years, College/university 4 years or more. These educational levels were categorized into three groups: “Primary education”, “Secondary education” and “University education”.

Living with spouse or partner was assessed by the question “Who do you live with?“, with one response option indicating “Spouse or partner”. Those indicating “partner/spouse” were categorized as “living with spouse or partner”, while non-response and responses indicating living with others were categorized as “no spouse or partner”. Body mass index (BMI) was categorized into three groups: <25, 25–30 and > 30 kg/m2. Furthermore, insomnia was evaluated using two specific questions: “How often during the last three months have you had difficulty falling asleep at night?” and “How often during the last three months have you woken up too early and couldn’t get back to sleep?” Response options were “Never/seldom,” “Sometimes,” and “at least three times a week.”. Participants who responded with “Never/seldom,” and/or “Sometimes,” were classified as having ‘no insomnia’. Those who responded “at least three times a week” to one or both questions were considered as having ‘insomnia’.

For smoking status, response options were “Never smoked,” “Former smoker,” “Smokes daily,” and “Smokes occasionally.” They were subsequently categorized into three smoking status groups: “Never smoked”, “Current smoker” and “Smoked previously”.

### Statistical analysis

Statistical analyses were conducted using SPSS version 28. Initially, descriptive statistics were used for all variables, including means and standard deviations for continuous variables and frequencies and percentages for categorical variables. To analyze the relationship between LTM and STM complaint scores and the independent variables, independent sample t-tests were utilized for dichotomous variables (sex, living with spouse or partner, insomnia, anxiety and depression) and one-way analyses of variance (ANOVA) for variables with more than two categories (age group, education level, BMI, smoking status and physical activity). LTM and STM problems were cross-tabulated with the independent variables for each time point. In the ANOVA pairwise comparisons, the Bonferroni correction was applied to explore significant differences between group means and adjust for inflating Type I error rates. Multivariable linear regression analysis was conducted to evaluate the associations between PA and LTM/STM complaint scores, while adjusting for potential confounders, including only variables demonstrating a relationship with *p* < 0.30 from the initial (unadjusted) analyses. Linear regression models were constructed for predicting LTM and STM complaint outcomes with HUNT3 and HUNT4 data, respectively. The models included age, sex, education level, living with spouse or partner, BMI, insomnia, anxiety, depression, smoking status, and PA as independent variables (provided *p* < 0.30 in bivariate analyses). In the analyses, all independent variables were included together, thus resulting in measures of associations adjusted for the influence of all other variables. In the final iteration of the analysis, relevant variables from HUNT3 were used in the longitudinal prediction of LTM/STM complaint scores observed in HUNT4, while also adjusting for the relevant LTM/STM complaint scores from HUNT3. Results with p-values < 0.05 were considered statistically significant.

### Ethics

This study received ethical approval from the Regional Committee for Medical Research Ethics (No. 624669). The HUNT was also approved by the Norwegian Data Inspectorate. Each participant in the HUNT surveys signed a written informed consent regarding the collection and use of data for research purposes. Participants can demand to have their data deleted from the HUNT database at any given moment. All research in HUNT is in accordance with the guidelines of the Regional Committee for Medical Research Ethics (REK), Data Inspectorate and applicable law. The study adhered to the Declaration of Helsinki.

## Results

### Sample characteristics

Table 1 presents the characteristics of participants in both the HUNT3 and HUNT4 studies. The baseline characteristics of participants in both samples were similar, with few major differences observed. Among the participants in HUNT4, a larger proportion reported to be former smokers, and a smaller proportion were current smokers, compared to participants in the HUNT3 dataset. In addition, while about 60% were classified with an intermediate PA level in both HUNT3 and in HUNT4, the HUNT4 participants had a smaller proportion of individuals with a low level of PA and a higher proportion with a high level of PA. Similar proportions were classified with low levels of anxiety (92%) and depression symptoms (95%-96%) in HUNT3 and HUNT4, respectively.

**Table 1:** Descriptive characteristics of the study participants in the two waves of data collection

Variables		HUNT3 (*n* = 9226)			HUNT4 (*n* = 6986)		
		n	%	M (SD)	n	%	M (SD)
Sex	Female	4850	52.6%		3706	53.0%	
	Male	4376	47.4%		3280	47.0%	
Age				59.3 (7.4)			69.3 (6.6)
Education	Primary education	-	-		1037	14.9%	
	Secondary education	-	-		3397	48.8%	
	University education	-	-		2520	36.2%	
Relationship status	No spouse/partner	2247	24.4%		1422	20.4%	
	Spouse/partner	6979	75.6%		5564	79.6%	
BMI [kg/m²]				27.0 (3.7)			26.8 (3.9)
Insomnia	No insomnia	6873	85.3%		5810	85.9%	
	Insomnia	1187	14.7%		956	14.1%	
Anxiety	Low anxiety	7293	92.1%		5385	91.9%	
	High anxiety	623	7.9%		474	8.1%	
Depression	Low depression	7549	94.9%		5670	95.6%	
	High depression	407	5.1%		258	4.4%	
Smoking status	No	3904	43.1%		2854	41.0%	
	Former	3398	37.5%		3603	51.7%	
	Yes	1757	19.4%		511	7.3%	
Physical activity	Low	2179	27.7%		1130	18.1%	
	Intermediate	4605	58.6%		3761	60.3%	
	High	1078	13.7%		1344	21.6%	

*LTM *refers to Long-Term Memory complaints, *STM *to Short-Term Memory complaints, and *BMI *to Body Mass Index

Valid % is reported. Missing data ranging between 0% and 16.1% for the variables. In the HUNT3 dataset, education level had too many missing values to be meaningfully employed

### Memory complaints in different subgroups

Table 2 presents the LTM and STM complaint ratings of the participants within subgroups for both waves of data collection. In HUNT3, and compared to their counterparts, higher LTM complaint ratings were shown for men, those aged 70–79, and those reporting insomnia and high levels of anxiety and depression. Consistent non-smokers had lower LTM complaint ratings than current and former smokers. Regarding STM complaints, higher ratings were shown for men, those who were overweight (BMI 25–30), those with insomnia, and those with high levels of anxiety and depression, compared to their counterparts. Consistent non-smokers had lower STM complaint ratings than current and former smokers, while those with low PA had higher STM complaint ratings than those with intermediate levels of PA.

**Table 2:** Subgroup differences in LTM and STM complaints in the two waves of data collection

Variables		HUNT3				HUNT4			
		LTM		STM		LTM		STM	
		M (SD)	p	M (SD)	p	M (SD)	p	M (SD)	p
Gender	Female	6.7 (1.8)	< 0.001	4.9 (1.2)	< 0.001	6.2 (1.2)	< 0.001	5.2 (1.7)	< 0.001
	Male	6.9 (1.8)		5.2 (1.4)		6.5 (1.3)		5.6 (1.7)	
Age	60–69	6.8 (1.8)	< 0.001	5.0 (1.3)	0.11	6.3 (1.3)	< 0.01	5.2 (1.7)	< 0.001
	70–79	7.2 (1.6)		5.1 (1.3)		6.2 (1.2)		5.5 (1.8)	
	80+	6.6 (2.0)		5.2 (1.4)		6.5 (1.3)		5.9 (1.9)	
Education level	Primary educ.	-		-		6.3 (1.3)	0.74	5.7 (1.9)	< 0.001
	Secondary educ.	-		-		6.3 (1.2)		5.4 (1.8)	
	University educ.	-		-		6.3 (1.2)		5.2 (1.6)	
Relationship status	No spouse	6.9 (1.8)	0.25	5.1 (1.3)	0.24	6.3 (1.3)	0.35	5.5 (1.8)	< 0.05
	Spouse	6.8 (1.7)		5.0 (1.3)		6.3 (1.2)		5.3 (1.7)	
BMI [kg/m²]	< 25	6.8 (1.7)	0.08	5.0 (1.3)	< 0.001	6.2 (1.3)	0.30	5.3 (1.7)	< 0.01
	25–30	6.8 (1.8)		5.1 (1.3)		6.3 (1.2)		5.4 (1.7)	
	> 30	6.7 (1.8)		5.0 (1.3)		6.3 (1.2)		5.5 (1.8)	
Insomnia	No insomnia	6.7 (1.7)	< 0.001	5.0 (1.3)	< 0.001	6.3 (1.2)	< 0.01	5.3 (1.7)	< 0.05
	Insomnia	7.4 (1.8)		5.3 (1.4)		6.4 (1.4)		5.5 (1.8)	
Anxiety	Low anxiety	6.7 (1.7)	< 0.001	5.0 (1.3)	< 0.001	6.3 (1.2)	< 0.05	5.3 (1.7)	< 0.001
	High anxiety	7.6 (1.8)		5.8 (1.5)		6.4 (1.3)		5.8 (1.8)	
Depression	Low depression	6.7 (1.7)	< 0.001	5.0 (1.3)	< 0.001	6.3 (1.2)	< 0.001	5.3 (1.7)	< 0.001
	High depression	7.8 (1.8)		6.0 (1.6)		6.6 (1.3)		6.2 (1.8)	
Smoking status	No	6.7 (1.8)	< 0.001	5.0 (1.3)	0.001	6.3 (1.3)	0.25	5.3 (1.7)	< 0.01
	Former	6.9 (1.7)		5.1 (1.3)		6.3 (1.2)		5.4 (1.8)	
	Yes	6.8 (1.7)		5.1 (1.3)		6.3 (1.2)		5.3 (1.7)	
Physical activity	Low	6.8 (1.7)	0.34	5.1 (1.3)	< 0.05	6.4 (1.3)	0.41	5.5 (1.8)	< 0.01
	Intermediate	6.8 (1.8)		5.0 (1.3)		6.3 (1.2)		5.3 (1.7)	
	High	6.7 (1.7)		5.0 (1.3)		6.3 (1.2)		5.4 (1.7)	

*LTM *refers to Long-Term Memory complaints, *STM *to Short-Term Memory complaints, and *BMI *to Body Mass Index

Valid % is reported. In the HUNT3 dataset, education level had too many missing values to be meaningfully employed

In HUNT4, higher LTM complaint ratings were shown for men, those over 80 years of age, those with insomnia, and those with high levels of anxiety and depression. STM complaint ratings were higher for men, increased linearly across age groups, and decreased linearly across levels of education. Compared to their counterparts, STM complaint ratings were also higher among those living without a spouse or partner, those with higher BMI, those with insomnia and those with higher levels of anxiety and depression. In addition, STM complaint ratings were higher among former smokers compared to consistent non-smokers, and higher among individuals with low levels of PA compared to those with intermediate and high levels.

### Adjusted associations with memory complaints

Table 3 presents the cross-sectional direct associations between the independent variables and LTM and STM complaints assessed in the HUNT3 study. Higher LTM complaint ratings were associated with male sex, higher age, insomnia, and high levels of anxiety and depression symptoms. There was also a relationship between smoking and higher LTM complaints. Higher STM complaint ratings were associated with the same variables.

**Table 3:** Linear regression analysis showing cross-sectional direct associations with LTM and STM complaints (HUNT3)

Variables	LTM (*n* = 7340)			STM (*n* = 6374)		
	B	95% CI	p	B	95% CI	p
Sex	0.25	0.18–0.33	< 0.001	0.38	0.32–0.44	< 0.001
Age (cont.)	0.01	0.03–0.04	< 0.001	0.01	0.00-0.01	< 0.001
Relationship status	0.05	-0.06-0.17	0.36	-0.03	-0.12-0.06	0.56
BMI [kg/m²] (cont.)	-0.01	-0.02-0.02	0.12	-0.00	-0.01-0.01	0.88
Insomnia	0.55	0.44–0.67	< 0.001	0.20	0.11–0.29	< 0.001
Anxiety	0.58	0.42–0.76	< 0.001	0.59	0.47–0.71	< 0.001
Depression	0.79	0.60–0.97	< 0.001	0.78	0.63–0.93	< 0.001
Smoking status	0.13	0.12–0.29	< 0.001	0.05	0.01–0.09	< 0.05
Physical activity	-	-	-	-0.03	-0.08-0.02	0.18
Explained variance		6.7%	< 0.001		7.1%	< 0.001

*LTM *refers to Long-Term Memory complaints, *STM *to Short-Term Memory complaints, and *BMI *to Body Mass Index

Cont. indicates continuous variables

Categorical variables are coded as follows: Sex (female is 0, male is 1), insomnia (no is 0, yes is 1), anxiety (low level is 0, high level is 1), depression (low level is 0, high level is 1), smoking status (non-smoker is 1, former smoker is 2, current smoker is 3), physical activity (low level is 1, intermediate level is 2, high level is 3)

Table 4 presents the cross-sectional direct associations between the independent variables and LTM and STM complaints assessed in the HUNT4 study. Higher LTM complaint ratings were associated with male sex, higher age, insomnia, and high levels of depression symptoms. Higher STM complaint ratings were associated with male sex, higher age, lower education level, in addition to higher levels of anxiety and depression.

**Table 4:** Linear regression analysis showing cross-sectional direct associations with LTM and STM complaints (HUNT4)

Variables	LTM (*n* = 2639)			STM (*n* = 4882)		
	B	95% CI	p	B	95% CI	p
Sex	0.32	0.23–0.42	< 0.001	0.45	0.35–0.55	< 0.001
Age (cont.)	0.01	0.00-0.02	< 0.05	0.03	0.02–0.04	< 0.001
Education level	-	-	-	-0.18	-0.25- -0.11	< 0.001
Relationship status	-	-	-	-0.08	-0.20-0.05	0.23
BMI [kg/m²] (cont.)	-	-	-	0.01	-0.01-0.02	0.40
Insomnia	0.15	0.03–0.28	< 0.05	0.08	-0.06-0.22	0.24
Anxiety	0.15	-0.01-0.31	0.07	0.43	0.25–0.62	< 0.001
Depression	0.29	0.09–0.48	< 0.01	0.57	0.31–0.82	< 0.001
Smoking status	0.03	-0.05-0.11	0.42	0.04	-0.04-0.12	0.33
Physical activity	-	-	-	-0.05	-0.12-0.03	0.25
Explained variance		2.7%	< 0.001		4.7%	< 0.001

*LTM *refers to Long-Term Memory complaints, *STM *to Short-Term Memory complaints, and *BMI *to Body Mass Index

Cont. indicates continuous variables

Categorical variables are coded as follows: Sex (female is 0, male is 1), education level (primary school is 1, secondary school is 2, university is 3), insomnia (no is 0, yes is 1), anxiety (low level is 0, high level is 1), depression (low level is 0, high level is 1), smoking status (non-smoker is 1, former smoker is 2, current smoker is 3), physical activity (low level is 1, intermediate level is 2, high level is 3)

Table 5 presents the direct prospective associations between independent variables assessed in HUNT3, and LTM and STM complaints assessed in the HUNT4 study. Higher LTM complaint ratings in HUNT4 were predicted by male sex and higher levels of LTM complaints 11 years earlier. Higher STM complaint ratings in HUNT4 were predicted by male sex, higher age, high levels of depression, and higher levels of STM complaints 11 years earlier.

**Table 5:** Linear regression analysis showing prospective associations between HUNT3 predictors and LTM and STM complaints 11 years later

Variables	LTM (*n* = 1443)			STM (*n* = 3146)		
	B	95% CI	p	B	95% CI	p
Sex	0.39	0.28–0.51	< 0.001	0.21	0.10–0.32	< 0.001
Age (cont.)	-0.01	-0.02-0.02	0.12	0.02	0.02–0.03	< 0.001
Relationship status	-0.10	-0.27-0.08	0.27	-0.16	-0.33-0.01	0.06
BMI [kg/m²] (cont.)	0.01	-0.01-0.02	0.43	0.01	-0.01-0.02	0.28
Insomnia	-0.02	-0.19-0.14	0.77	-0.08	-0.25-0.08	0.32
Anxiety	-0.06	-0.28-0.16	0.60	-0.13	-0.36-0.10	0.28
Depression	0.16	0.09–0.41	0.20	0.31	0.04–0.59	< 0.05
Smoking status	0.02	-0.06-0.10	0.56	0.07	0.00-0.15	0.05
Physical activity	-0.05	-0.14-0.05	0.32	-0.04	-0.12-0.05	0.37
LTM	0.33	0.30–0.37	<0.001	-	-	-
STM	-	-	-	0.52	0.48–0.57	< 0.001
Explained variance		20.8%	< 0.001		17.6%	< 0.001

*LTM *refers to Long-Term Memory complaints, *STM *to Short-Term Memory complaints, and *BMI *to Body Mass Index

Cont. indicates continuous variables

Categorical variables are coded as follows: Sex (female is 0, male is 1), insomnia (no is 0, yes is 1), anxiety (low level is 0, high level is 1), depression (low level is 0, high level is 1), smoking status (non-smoker is 1, former smoker is 2, current smoker is 3), physical activity (low level is 1, intermediate level is 2, high level is 3)

### Attrition analysis

Finally, the sample used in the longitudinal analysis was compared with the initial sample at baseline (HUNT3). Gender differences were not statistically significant, but compared with the HUNT3 sample, the sample used in the longitudinal analysis were younger (M = 57.9 years vs. 61.1 years, *p* < 0.001), had lower BMI (M = 26.7 vs. 27.3, *p* < 0.001), had a higher proportion of participants living with a spouse or partner (77.5% vs. 73.3%, *p* < 0.001), and a lower proportion with insomnia (12.5% vs. 17.6%, *p* < 0.001), anxiety (6.7% vs. 9.3%, *p* < 0.001) and depression (4.3% vs. 6.1%, *p* < 0.001). This sample were less inclined to be smokers (17.9% vs. 21.3%) and had a higher proportion who had never smoked (44.9% vs. 40.8%, *p* < 0.001) and were more inclined to have high levels of PA, compared to their counterparts (14.7% vs. 12.5%, *p* < 0.01).

## Discussion

The aim of this study was to assess possible relationships between PA, MH and concurrent and subsequent memory complaints among adults and older adults. While initial subgroup comparisons showed higher levels of STM and LTM complaints among those with lower levels of PA, this association was no longer significant – neither cross-sectionally, nor longitudinally – after adjustment for sociodemographic and MH-related variables. Instead, male gender and poorer MH, particularly higher levels of depressive symptoms, stand out as factors associated with concurrent and subsequent STM and LTM complaints.

Our study found that poorer MH, especially higher levels of depressive symptoms, is linked to current and future memory complaints in older adults. Depression, anxiety, and apathy are common among individuals with mild cognitive impairment, and evidence suggests that the combined impact of these psychopathological symptoms may accelerate cognitive decline and increase the risk of developing dementia [41, 58]. Furthermore, clinical studies have shown that patients with mild cognitive impairment have higher rates of depression [59], have worse memory functioning [60, 61], and have higher conversion rates to dementia [41]. This aligns with research showing that depression is associated with vascular changes in the brain, such as white matter hyperintensities, which are linked to cognitive decline [62, 63]. Importantly, previous research has demonstrated that subjective memory complaints are only weakly related to objective memory impairment. Our findings suggest that depressive symptoms may influence how individuals perceive their memory functioning, contributing to greater subjective complaints irrespective of any objective memory deficits. This highlights an important opportunity: subjective memory complaints may serve as early warning signs for cognitive decline and be a valuable target for timely intervention, and attention among healthcare personnel towards older adults’ perceived memory problems may be particularly important in the case of previous or concurrent depressive symptoms.

Our results suggest a directional influence from MH to subjective memory complaints and potentially to later memory function, rather than these factors merely reflecting simultaneous manifestations of underlying brain changes. Beyond biological mechanisms, depression can affect cognition through reduced social engagement. Depressed individuals often experience social withdrawal and lowered motivation, leading to participation in fewer mentally and physically stimulating activities—behaviors that are essential for maintaining brain health [21]. This reduced engagement may worsen subjective memory complaints and accelerate cognitive decline in the long run. Conversely, memory performance, even as a subjective perception, is central to personal identity and social functioning. Self-perceived and objective declines in memory lead to increased dependency, social isolation, and MH problems such as psychological distress, depression, and anxiety [5, 35, 36]. Therefore, improving MH and promoting socially and physically active lifestyles may help protect memory in older adults. Moreover, subjective memory complaints may serve as an early warning signal, offering a valuable opportunity for timely intervention.

In our study, higher level of subjective memory complaints was observed among males. Previous research shows mixed findings regarding gender differences in cognitive performance and memory impairment [42, 44, 45, 64, 65]. Higher levels of subjective memory complaints in males likely reflect a combination of biological, lifestyle, and sociocultural factors. Biologically, males and females differ in brain structure and function; for example, females tend to have a larger and more active hippocampus, which is crucial for memory, and benefit from the neuroprotective effects of estrogen [66–68]. Gender-related differences in cognitive abilities, including the types of information men and women remember best, may also contribute [44].

However, non-biological factors may also contribute significantly. Males experience higher rates of cardiovascular disease, substance use, and other health conditions that negatively affect brain functioning, while also being less likely to seek medical care or engage in cognitively protective activities. Educational background, occupational exposure, and levels of cognitive stimulation may differ by sex and influence memory resilience. Sociocultural norms might also play a role as men may underreport early cognitive symptoms. This introduces the possibility of measurement bias and suggests that the prevalence of memory complaints among men may be even higher than observed in this study, highlighting the need to target this group with specific interventions to support cognitive health and prevent further decline. Taken together, these biological vulnerabilities, environmental influences, and sociocultural factors help explain why men in our study reported higher scores on subjectively experienced memory complaints, even if objective memory performance does not always reflect these self-reports [16].

Surprisingly, although individuals with lower levels of PA reported more STM complaints in both HUNT3 and HUNT4, these associations were no longer significant after adjusting for sociodemographic and mental health-related variables. No significant associations were observed between PA and LTM complaints either. Our findings are consistent with those of some previous studies [20, 26, 69], suggesting weak or non-existing associations between PA and subjective memory complaints. However, they contrast with the findings of other studies [24, 25, 70]. More specifically, some studies focusing on aerobic exercise have reported that higher levels of aerobic activity are more strongly linked to improvements in LTM than STM complaints [7, 23, 71]. However, those studies focused on acute or specific PA intensities and did not account for long-term habitual PA, limiting the comparability to our findings. The divergence between our results and previous studies may also reflect methodological differences, particularly in how PA and memory complaints were measured, as well as differences in study populations and designs.

Given the 11-year interval between data collection waves, our findings do not support the idea that the PA during mid-adulthood contribute to building better “cognitive reserve” with long-lasting benefits on subjectively perceived memory—whether short-term or long-term. This discrepancy may be due to differences in study design, follow-up duration, population characteristics, or measurement approaches. Although our observational design limits causal inferences, the consistency of associations between MH - particularly depressive symptoms - and memory complaints over time suggests a robust and enduring influence. This highlights the importance of addressing MH in adult and older adult populations as a potentially modifiable factor that may have implications for the concurrent or later experience of cognitive concerns. Nevertheless, research has shown that higher levels of PA decrease the risk of anxiety [72], and reduce the risk of depression in children, adults and older adults [73]. Similarly, an overview of systematic reviews has demonstrated the substantial benefits of PA in reducing symptoms of depression, anxiety, and psychological distress across diverse adult populations—including the general population, individuals with diagnosed MH conditions, and those with chronic illnesses—highlighting its significant therapeutic potential [74]. Thus, regardless of any direct effect on subjective memory complaints, PA should be encouraged due to its numerous benefits, including MH.

### Study strengths and limitations

This study has several notable strengths. First, it utilizes data from the large and well-established HUNT Study, which offers a robust population-based sample of older adults. The HUNT cohort enhances the generalizability of the findings to the broader Norwegian population, and potentially to other populations with similar demographic profiles. The inclusion of both cross-sectional and longitudinal analyses provides a more nuanced understanding of how PA and MH relate to both concurrent and future STM and LTM complaints. Furthermore, the study controls for a broad range of relevant confounders, including gender, age, and mental health-related variables, thereby strengthening the validity of the observed associations. By examining both STM and LTM complaints specifically, the study also contributes important depth to the literature on subjective memory decline in older adults.

Nevertheless, several limitations should be acknowledged. The primary limitation is the reliance on self-reported measures for PA, MH symptoms, and STM and LTM complaints, which may introduce bias due to recall inaccuracies or subjective interpretation. Previous research has shown that the correlation between subjective memory complaints and objective memory impairment is generally weak [16]. However, as noted earlier, subjective memory complaints can serve as an early indicator of cognitive changes, providing valuable information for identifying individuals at risk and guiding early interventions. Additionally, the PA assessment in the questionnaire refers only to activity over a typical week and does not distinguish between long-term and recent engagement in exercise. As a result, individuals who recently adopted active lifestyles may be grouped with those who have exercised consistently for years, potentially affecting the interpretation of the findings. However, the use of self-reported measures also offers strengths, such as the ability to capture data from a large, naturalistic sample without altering participants’ everyday routines, which could influence PA behavior and MH. Moreover, self-report is a widely used method in epidemiological studies examining PA, MH and cognitive function in aging populations [29, 75]. While the study adjusted for several key variables, we did not have access to disease-specific risk factors for dementia, such as stroke and hypertension [21], and the impact of such risk factors on LTM and STM complaints in the current study is therefore unknown. The cross-sectional design of the primary analyses restricts causal interpretations, and further studies are needed to establish causal relationships. Although longitudinal data were included, these analyses were limited by a substantial loss of data as the analyses required valid responses on each included variable. As demonstrated by the attrition analysis, the longitudinal analyses were performed on a younger and healthier subsample, compared to the initial sample assessed at baseline (HUNT3), which limits the possibility to generalize the longitudinal associations to the general population. Other relevant factors—such as medication use and social connectivity—were not assessed and may also impact both MH and memory performance.

### Implications for practice and further research

The persistence of depressive symptoms as a predictor in both cross-sectional and longitudinal analyses highlights the critical need to address MH as a modifiable risk factor for SMI. SMI may serve as an early warning sign for cognitive decline, and the strong association with depressive symptoms suggests that intervening in MH could have protective effects on memory function. In practice, this underscores the importance of routine screening for depressive symptoms in individuals reporting memory concerns, as well as the integration of targeted interventions. Similarly, the identification of smoking as a longitudinal predictor of poorer STM complaints reinforces the importance of public health strategies targeting modifiable lifestyle behaviors to support cognitive health in older adults. While the direct and independent effect of PA on subjective memory complaints appears limited in this population-based sample, PA remains strongly recommended for healthy aging due to its well-established physical and psychological benefits—particularly its positive influence on MH, including the prevention and reduction of depressive symptoms. Moreover, older males may require closer monitoring for memory complaints, not only because they are often less likely to report symptoms, but also because they appear to experience higher levels of SMI. Early recognition and targeted support for this group could play an important role in mitigating cognitive decline.

Future studies should investigate whether interventions aimed at improving MH, particularly preventing and reducing depressive symptoms, could help mitigate memory complaints and possibly delay or prevent the onset of more serious cognitive impairments. Incorporation of objective neuropsychological testing, neuroimaging, and biological markers (e.g., vascular health, hippocampal volume) to better understand the mechanisms linking depression and memory. It would also be valuable to explore the role of apathy and anxiety in more detail, given their growing recognition as early indicators of cognitive decline. Finally, future research should aim for more diverse and representative samples to ensure generalizability across populations.

Future research should use objective measures of PA in real-time settings to better understand its impact on STM and LTM complaints. Ideally, randomized controlled trials will be conducted to establish causality. These studies should examine how different levels and types of PA influence the encoding, consolidation, and retrieval processes associated with STM and LTM complaints over both immediate and extended timeframes.

## Conclusion

This population-based study found no independent association between PA and SMI after controlling for sociodemographic and mental health-related variables. Instead, depressive symptoms consistently emerged as the strongest predictor of both STM and LTM complaints, both concurrently and, to an extent, over time. Male sex also stood out as a significant and persistent factor associated with higher levels of memory complaints, highlighting older men as a potentially underserved and at-risk group. These findings point to the need for greater clinical awareness and proactive monitoring of memory complaints among those with higher levels of depressive symptoms and among older men, who may be less likely to seek help yet more likely to experience SMI.

While PA did not show a direct effect on memory complaints, its established benefits for MH—especially in reducing depressive symptoms—underscore its continued importance for healthy aging. Public health strategies that prioritize MH support and target modifiable risk factors such as smoking and depressive symptoms. Future research should explore whether improving MH—particularly reducing depressive symptoms, apathy, and anxiety—can mitigate memory complaints and delay cognitive decline, using diverse and representative samples. Incorporating neuropsychological testing, neuroimaging, and biomarkers will help clarify the mechanisms linking MH with STM and LTM complaints.

## Data Availability

The datasets used and/or analyzed during the current study are available on reasonable request. Requests to access these datasets should be directed to Trøndelag Health Study (HUNT) (https://www.ntnu.edu/hunt/research).

## Abbreviations

MH: Mental health
PA: Physical activity
STM: Short–term memory
LTM: Long–term memory
HUNT study: Trøndelag Health Study in Norway
